# Genome-scale modeling and transcriptome analysis of *Leuconostoc mesenteroides* unravel the redox governed metabolic states in obligate heterofermentative lactic acid bacteria

**DOI:** 10.1038/s41598-017-16026-9

**Published:** 2017-11-16

**Authors:** Lokanand Koduru, Yujin Kim, Jeongsu Bang, Meiyappan Lakshmanan, Nam Soo Han, Dong-Yup Lee

**Affiliations:** 1 Department of Chemical and Biomolecular Engineering, National University of Singapore, 4 Engineering Drive 4, Singapore, 117576 Singapore; 2 Brain Korea 21 Center for Bio-Resource Development, Division of Animal, Horticultural, and Food Sciences, Chungbuk National University, Cheongju, Chungbuk 28644 Republic of Korea; 3 Bioprocessing Technology Institute, Agency for Science, Technology and Research (A*STAR), 20 Biopolis Way, #06-01, Centros, Singapore, 138668 Singapore

## Abstract

Obligate heterofermentative lactic acid bacteria (LAB) are well-known for their beneficial health effects in humans. To delineate the incompletely characterized metabolism that currently limits their exploitation, at systems-level, we developed a genome-scale metabolic model of the representative obligate heterofermenting LAB*, Leuconostoc mesenteroides* (*i*LME620). Constraint-based flux analysis was then used to simulate several qualitative and quantitative phenotypes of *L. mesenteroides*, thereby evaluating the model validity. With established predictive capabilities, we subsequently employed *i*LME620 to elucidate unique metabolic characteristics of *L. mesenteroides*, such as the limited ability to utilize amino acids as energy source, and to substantiate the role of malolactic fermentation (MLF) in the reduction of pH-homeostatic burden on F_0_F_1_-ATPase. We also reported new hypothesis on the MLF mechanism that could be explained via a substrate channelling-like phenomenon mainly influenced by intracellular redox state rather than the intermediary reactions. Model simulations further revealed possible proton-symporter dependent activity of the energy efficient glucose-phosphotransferase system in obligate heterofermentative LAB. Moreover, integrated transcriptomic analysis allowed us to hypothesize transcriptional regulatory bias affecting the intracellular redox state. The insights gained here about the low ATP-yielding metabolism of *L. mesenteroides*, dominantly controlled by the cellular redox state, could potentially aid strain design for probiotic and cell factory applications.

## Introduction

Obligate heterofermentative lactic acid bacteria (LAB) lacking the glycolytic enzyme, fructose 1,6 bisphosphate aldolase, dissimilate carbon solely through a unique route of glycolysis known as the phosphoketolase pathway (PKP)^1^. This pathway is fundamentally different from the canonical Embden-Meyerhof-Parnas (EMP) pathway wherein one mole of glucose-6-phosphate is broken down into two moles of glyerone-3-phosphate at the end of the preparatory phase. In PKP, on the other hand, one mole of glucose-6-phophate is converted into one mole of each acetyl-phosphate and glyerone-3-phosphate via phophoketolase, thus leading to mixed fermentation products such as acetate, lactate and ethanol. These properties along with their natural ability to degrade xylan from lignocellulose using endogenous β-xylosidases^2^ make obligate heterofermentative LAB (e.g. *Leuconostoc mesenteroides* and *Lactobacillus fermentum*) one of the dominant species in vegetable fermentations such as Korean kimchi and sauerkraut^3,4^. The heterofermentative by-products inhibit the growth of harmful microbes and simultaneously impart taste and flavour to the final food products. In addition, the obligate heterofermentative LAB exhibit several beneficial characteristics to mammalian hosts including antimicrobial activities^5–7^, immunomodulatory effects^8,9^ and antioxidant activities^6,10^. They are also known to improve the microfloral balance in gastrointestinal tract; for example, *Lb. fermentum* is shown to strengthen it possibly through the increased activity of α-glycosidases and esterases^11^. In a different study, it is reported that a *L. mesenteroides* strain produces the prebiotic isomaltooligosaccharides that in turn enhance the growth of *Bifidobacterium* and *Lactobacillus* species in poultry chicken^12^.

However, despite their probiotic abilities and generally regarded as safe (GRAS) status, therapeutic applications of obligate heterofermentative LAB remain largely unexplored. The fastidious growth requirements and low ATP yield associated with the utilization of PKP are considered as the key bottlenecks for their practical applications. Interestingly, the heterofermentative LAB have naturally evolved to meet the cellular energy requirements under specific conditions by using alternate routes comprising (i) malolactic fermentation (MLF)^13^, (ii) alternate electron acceptors, e.g., fructose that yields mannitol upon reduction^14^, and (iii) phosphotransferase system (PTS) mediated transport^1,15^. Nonetheless, the underlying molecular mechanisms and their contributions to ATP yield, pH, and redox states still remain elusive. Elucidating such metabolic routes related to the unique cellular phenotype of LAB is, therefore, integral to their successful probiotic application^16^. For example, metabolic determinants restoring cofactor balance can be identified to modulate the relative abundance of other probiotic bacteria in the gut for imparting beneficial health effects^17,18^. To this end, a systems biology approach based on *in silico* metabolic modeling and omics data integration could greatly aid to decipher the lesser investigated metabolic landscapes of these LAB.

Constraint-based flux analysis, also known as flux balance analysis (FBA), is one of the well-established approaches for investigating cellular metabolism at systems-level under various environmental/genetic perturbations^19^. The availability of software applications to conveniently implement FBA and related *in silico* methods^20^ has enabled the development of genome-scale models (GEMs) for more than 100 species across all three domains of life, thereby facilitating analysis of their intracellular metabolism^21^. However, with respect to LAB, only a handful of GEMs are available, including *Lactococcus lactis* ^22–24^, *Lactobacillus plantarum* ^25^, *Streptococcus thermophilus* ^26^ and *Lactobacillus casei* ^27,28^ although more than 100 LAB genomes have been sequenced^29^. Note that these species with available GEMs belong to either homolactic or facultatively heterolactic fermenting groups. To enable the systems analysis of the other important group, i.e., obligate heterofermentative, herein, we present new genome-scale metabolic reconstruction of *Leuconostoc mesenteroides* ssp. *mesenteroides* ATCC 8293^30^. The reconstructed model was subsequently validated both qualitatively and quantitatively using the fermentative substrate phenotyping and batch culture data, respectively. The model was also used together with transcriptome data for generating new hypotheses related to energy and redox metabolisms, and their effects on cellular phenotype.

## Results

### Reconstruction of *L. mesenteroides* genome-scale metabolic network

**Figure 1:**
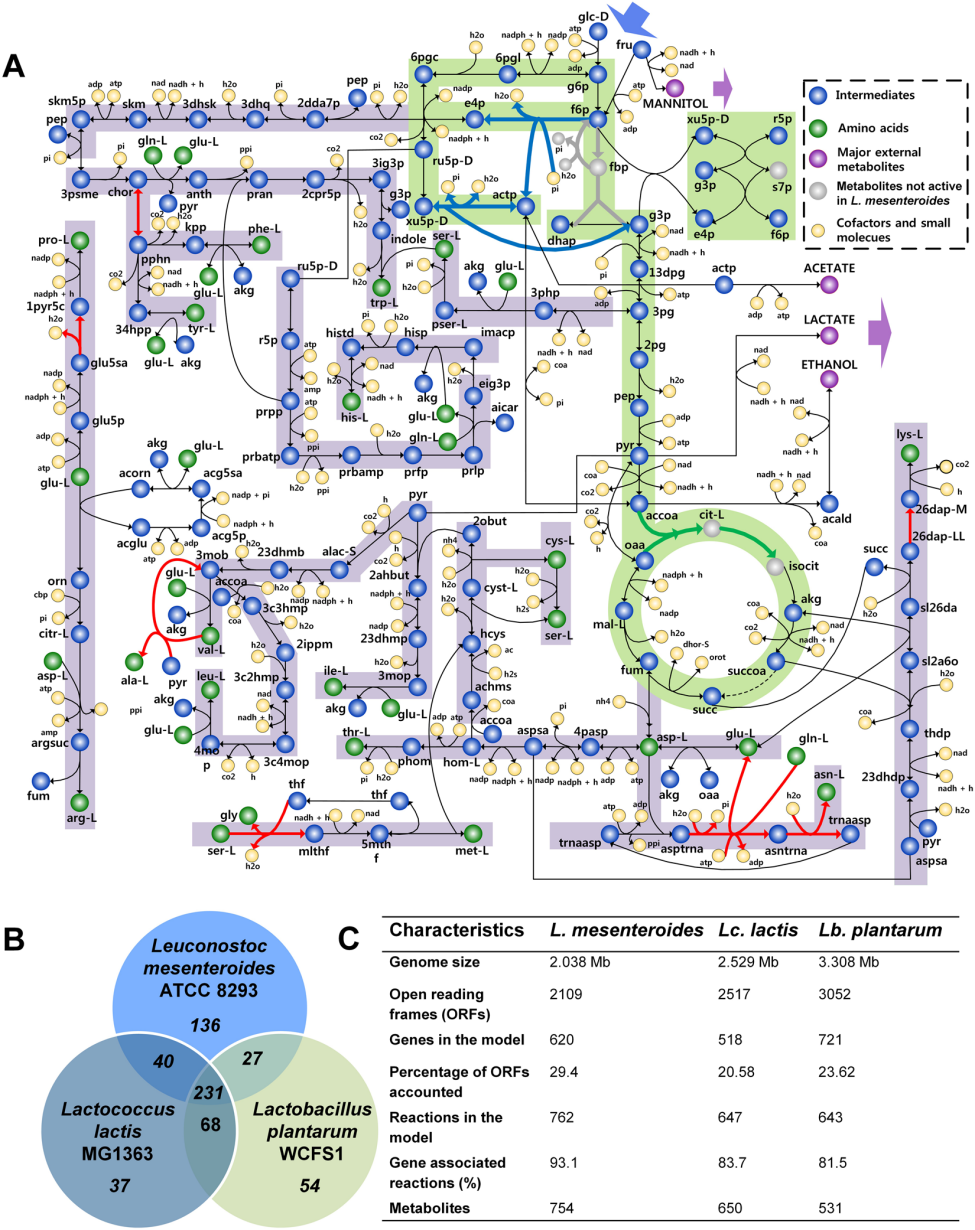
*L. mesenteroides* metabolic network (iLME620) reconstruction and comparison of its characteristics with other LAB. (**A**) Central metabolism of *L. mesenteroides* along with amino acid biosynthetic pathways present in *i*LME620. Reactions represented by ‘red’ lines are added to the network during the manual curation process. Reactions in ‘grey’ represent those absent in *L. mesenteroides*, but present in the other two LAB; reactions in ‘blue’ represent those present in *L. mesenteroides* and *Lb. plantarum*, but absent in *Lc. lactis*; reactions in ‘green’ represent those absent in *L. mesenteroides* and *Lb. plantarum*, but present in *Lc. lactis*; reaction with dotted line is absent in all three LAB (**B**) Venn diagram showing comparison of EC numbers of *i*LME620 with the GEMs of *Lactococcus lactis* subsp. cremoris MG1363 and *Lactobacillus plantarum* WCFS1. (**C**) Comparison of metabolic network characteristics of the LAB.

The genome-scale metabolic network of *L. mesenteroides* was newly reconstructed following established procedures^31^ (see Methods). Initially, the draft metabolic network was built based on information obtained from the genome annotation^32^ and biochemical databases such as MetaCyc^33^, KEGG^34^ and TransportDB^35^. Subsequently, the metabolite dead-ends were identified using GapFind algorithm^36^; new metabolic reactions supported by experimental and literature evidences were added to fill the network gaps. Reaction directionality was assessed using the available thermodynamic data calculated at physiologically relevant pH and intracellular metabolite concentrations as described earlier^37–39^ (see Methods). We then used this draft model to explore the minimal nutrient requirements of *L. mesenteroides* by mimicking the single nutrient-omission experiments conducted using a chemically defined medium (CDM)^40^. The comparison of *in silico* predictions with the experiments revealed some discrepancies which were then resolved through further manual curation of the network model. For example, the amino acid auxotrophy experiments confirmed asparagine as one of the non-essential amino acids. However, initial simulation of the draft model gave rise to no growth in the absence of asparagine, as the common asparagine biosynthetic enzymes, i.e., EC 6.3.5.4 and EC 6.3.1.1, were absent. From the genome-annotation evidence, we found the presence of an unique enzyme, aspartyl-tRNA-asparagine amidotransferase (EC 6.3.5.6) in *L. mesenteroides*, which indicated the possible existence of a non-canonical route for the biosynthesis of asparagine using aspartyl-tRNA and glutamine as substrates^41^. Similarly, vitamin-auxotrophy data was also used to further enhance the model quality. Folate, an indispensable precursor in the biosynthesis of DNA, was found to be only stimulatory according to the experiments. On contrary, the *in silico* simulations require its external supply for the cell growth, indicating possible missing annotations in the folate biosynthetic pathway. Therefore, a few non-gene associated reactions were added into the reconstruction in order to complete their biosynthetic pathways. BLASTp homology searches were then performed for the corresponding enzymes which were newly added, thereby substantiating their inclusion. Overall, the curated model was able to predict the auxotrophy of 7 out of 8 vitamins and 19 out of 20 amino acids accurately (Supplemental data 1). A central metabolic pathway map showing the biosynthetic pathways of various amino acids in *L. mesenteroides* is depicted in Fig. 1A. The list of reactions added during the gap-filling process is provided in Supplemental data 1.

The resultant genome-scale metabolic network of *L. mesenteroides*, *i*LME620, includes 620 genes, 754 metabolites, 762 metabolic and transport reactions, and one reaction representing the biomass assembly (see Methods). We measured the major elemental compositions of *L. mesenteroides* biomass and compared them with the molar elemental constituents of the biomass equation which is derived from the assembly of the measured macromolecular compositions (Supplemental data 1). The values in the equation are within ± 20% range of the experimental measurements (C H_1.765_ N_0.18_ O_0.53_ compared to that of C H_2.05_ N_0.197_ O_0.455_). The complete list of reactions, metabolites and associated genes accounted in *i*LME620, and the calculations involved in the biomass equation assembly are provided in Supplemental data 1. The model is also available as Systems Biology Markup Language (SBML) file (level 3, version 1, http://sbml.org/) in Supplemental data 2.

We compared *i*LME620 with the GEMs of other LAB, *Lb. plantarum* ^25^ and *Lc. lactis* ^24^, using the EC numbers in order to gain insights into their unique metabolic features (Fig. 1A–C). Expectedly, significant differences were observed in the preparatory phase of glycolysis, the non-oxidative branch of pentose phosphate pathway and the initial steps of the tricarboxylic acid cycle (Fig. 1A). We also found that the unique EC numbers of *Lb. plantarum* and *Lc. lactis* mostly fall under central metabolism and biomass biosynthetic pathways. Interestingly, most of the unique EC numbers in *L. mesenteroides* are unclassified and belong to the family of oxidoreductases hinting at a possible dominant redox metabolism (Supplemental Fig. 1). We further evaluated the ability of three LAB to grow under various environmental perturbations, e.g., amino acid auxotrophy and sugar utilization (see Methods and Supplemental Fig. 2). *L. mesenteroides* is auxotrophic to relatively less number of amino acids and can additionally utilize xylose as carbon source, which is a common characteristic of microbes associated with plant-based ecological niches^42,43^. We also examined the distribution of essential genes in three LAB, resulting in similar overall trends across several pathways with exceptions in certain cofactor biosynthetic pathways (see Methods and Supplemental Fig. 3).

**Figure 2:**
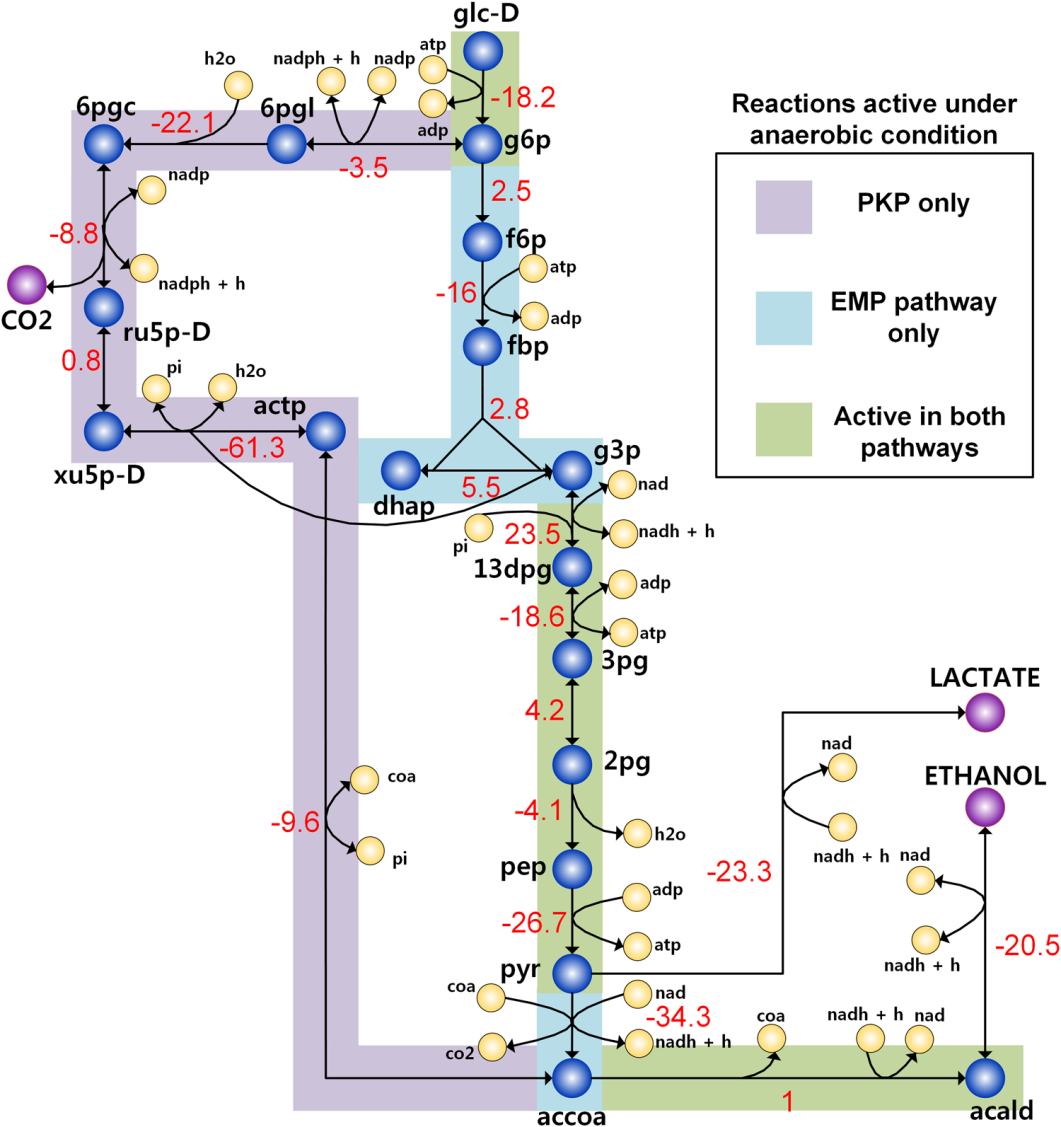
Comparison of Gibbs free energy changes of reactions under anaerobic condition in PKP and EMP pathway. The transformed Gibbs free energy changes of reactions (highlighted in red color) in the pathways were calculated using eQuilibrator^73^, for pH 7.2, ionic strength (0.1 M) and intracellular metabolite concentrations (1μM-10mM). For expanded names of all metabolites (black) and reactions (blue) (except, PFK: phosphofructokinase and FBA: Fructose bisphosphate aldolase, which are absent in *L. mesenteroides*), see Supplemental data 1.

A detailed comparison of glycolytic pathways shows that while both *Lb. plantarum* and *Lc. lactis* possess high ATP-yielding EMP pathway (2 moles of ATP per mole of glucose), the low ATP-yielding PKP (one mole of ATP per mole of glucose) is the only route of carbohydrate catabolism in *L. mesenteroides* (Fig. 2). Notably, it has been reported that the significant loss of ATP yields is often, at least partially, compensated by their favourable thermodynamics, i.e., larger net negative standard Gibbs free energy change values (Δ_r_ *G*′°)^44,45^. Here, we demonstrated a similar thermodynamic advantage of PKP over the EMP pathway by computing Δ_r_ *G*′° of each reaction in either pathways as well as the net Δ_r_ *G*′° for the pathways at physiologically relevant anaerobic condition (see Methods). The results showed that PKP has a larger fraction of reactions with negative Δ_r_ *G*′° values (Fig. 2 **)** and a lower net Δ_r_ *G*′° (−186.8 kJ/mol in PKP vs −178.0 kJ/mol in EMP pathway), highlighting thermodynamic advantage of PKP over EMP pathway.

### Qualitative and quantitative validations of *in silico* model predictions

In order to validate the qualitative model predictions, we simulated fermentable substrate phenotyping for various carbon sources and compared the results with experimental literature^46,47^. The model predictions showed good agreement for 25 of the total 29 substrates tested. Here, the model was structured in a way that the glycoside substrates among the tested carbon sources are hydrolyzed by their respective extracellular glycosidases before the metabolizable portion of the molecule becomes available for its uptake. The nature of transport of these substrates, the enzymes involved and corresponding gene annotations, if any, are provided in Supplemental data 1.

**Figure 3:**
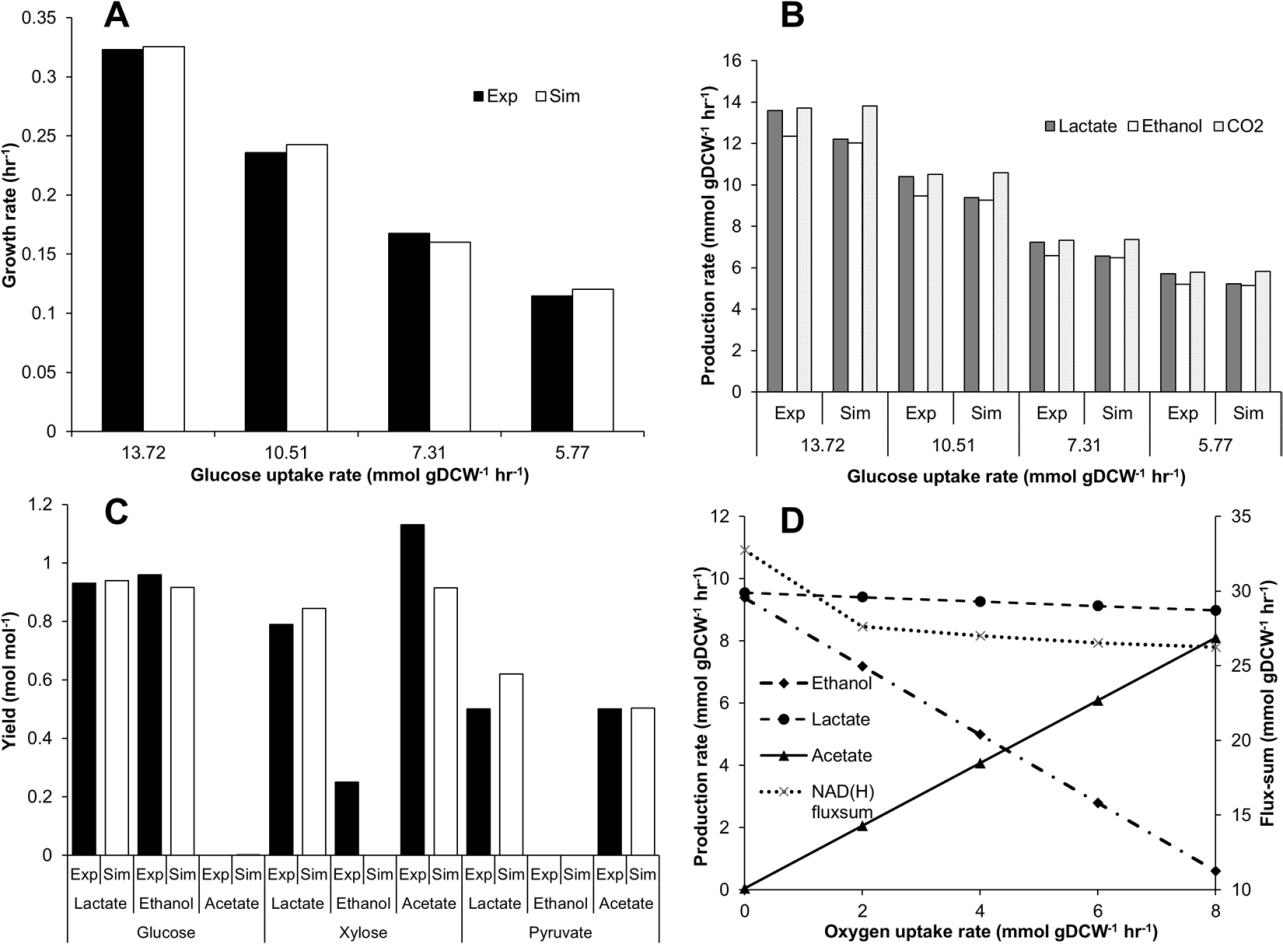
Quantitative predictions of *i*LME620. (**A**) Comparison of batch growth rates and *in silico* model predictions. (**B**) Comparison of batch fermentation end-product profiles and *in silico* model predictions; Exp: Experimental value, SIM: *In silico* simulation. (**C**) Comparison of *in silico* and experimentally observed by-product yields from glucose, xylose and pyruvate. (**D**) Effect of oxygen on the fermentative product profiles and the overall NAD(H) turnover rate as computed by flux-sum. Note that glucose uptake rate was constrained at 10 mmol gDCW^−1^ hr^−1^ for simulations used in Fig. 3C and D.

In addition, batch growth data from literature^48^ was used to evaluate model predictions, quantitatively. Exponential growth phase data points were used for the *in silico* predictions based on constraint-based flux analysis (see Methods). In order to mimic the complex medium condition, the uptake rates of all amino acids, vitamins and nucleotides were left unconstrained during the simulations. The values of growth associated maintenance (GAM) and non-growth associated maintenance (NGAM) were constrained at 13.613 mmol gDCW^−1^ and 1.104 mmol gDCW^−1^ hr^−1^, respectively. These energetic parameters were estimated as described earlier^25^ (Supplemental data 1). The comparison of *in silico* predicted growth rate and product formation profiles were highly consistent with the experimental data (Fig. 3A and B). We also simulated the growth using different carbon sources including glucose, xylose and pyruvate; the resulting by-product yields when compared to those reported in literature^49–51^ were found to be fairly consistent with the observed values (Fig. 3C). It should be highlighted that the growth simulations using *i*LME620 showed a limited uptake of all exogenously supplied amino acids, mostly proportional to the biomass protein demand as revealed by fluxes through reactions catalysed by the corresponding amino-acyl tRNA synthetases (Supplemental data 1). In contrast, the growth simulations on other LAB models determined unrealistically high amino acid uptake rates, which is attributed to the utilization of some of the supplied amino acid as sources of energy during biomass maximization. Furthermore, the model simulated with and without excess amino acid supply showed no differences in ATP yield (1 mole ATP per mole glucose in both scenarios) which is an unique characteristic of *L. mesenteroides*. This clearly suggests that *L. mesenteroides* has restricted or no capability of utilizing amino acids as energy source, invariably requiring regular carbon sources to derive energy. It should be noted that the relatively higher uptake rates of aspartate and glutamate or glutamine (Supplemental data 1) are to satisfy the additional demands imposed by peptidoglycan biosynthetic precursors, D-alanine and glucosamine-6-phosphate. Collectively, the qualitative and quantitative model validation results presented here clearly demonstrated high fidelity of the current model, *i*LME620.

**Figure 4:**
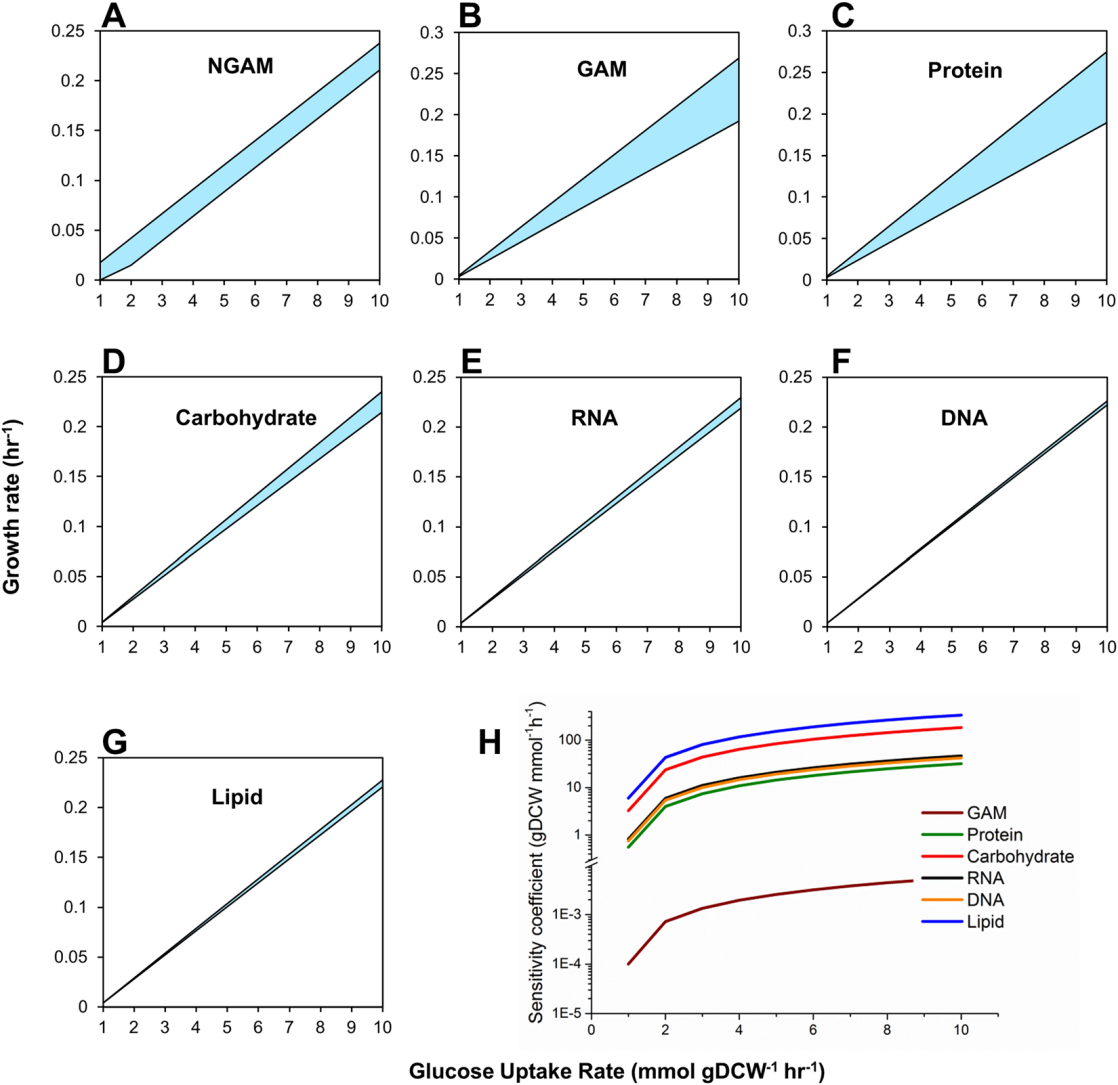
Sensitivity of cellular growth upon the changes in maintenance costs and macromolecular compositions. (**A**–**G**). Effect of 50% variation in the stoichiometries/composition of NGAM, GAM, Protein, Carbohydrate, RNA, DNA and Lipid, respectively on growth at different glucose uptake rates. (**H**) Plot of sensitivity coefficients, which are ratios of change in growth for unit changes in components for biomass equation, including GAM, Protein, Carbohydrate, RNA, DNA and Lipid, at different glucose uptake rates.

We also performed sensitivity analysis to evaluate the robustness of the model predictions, which can be affected by several fixed parameters such as the maintenance energy costs and stoichiometric coefficients of the individual metabolites in the biomass equation (see Methods). The effect of change in energy costs and macromolecular compositions on cellular growth was investigated, thereby identifying protein and GAM as critical components whose variations may affect the model prediction (Fig. 4A–G). We further accounted for potential errors in the measurement of each component by determining the change in growth objective to unit change in the stoichiometry of each component (see Methods), and observed that biomass is highly sensitive to lipid and least sensitive to protein measurements (Fig. 4H **)**. Hence, we should carefully estimate the energetic costs and potential errors in quantifying cellular lipid content, as these may vary across different growth environments.

### Intracellular redox state is a key factor determining the fermentative product profiles

The current model, *i*LME620, was used to elucidate metabolic states leading to the unique heterolactic fermentative behavior. For example, constraint-based flux simulations showed that lactic acid production during the anaerobic growth conditions is highly modulated by the tight coupling between NAD/NADH production and regeneration routes (Fig. 1A). Under aerobic growth condition, *L. mesenteroides* utilizes pyruvate oxidase in combination with NADH peroxidase to regenerate additional redox equivalents. Availability of such alternate routes diverts significant amounts of flux away from alcohol dehydrogenase towards pyruvate oxidase and acetate kinase. Hence, as the O_2_ uptake rate increases from zero to maximum, the yield of lactate, ethanol, and acetate changes from a molar proportion of 1:1:0 to 1:0:1, recapitulating the experimentally observed trend^49^ (Fig. 3D). This increase in acetate production further results in additional ATP synthesis leading to higher growth rate. The step decrease in the NAD(H) turnover rate with the supply of oxygen (Fig. 3D **)**, as quantified by flux-sum analysis^52^, indicates the plausible switch of pyruvate catabolism from alcohol dehydrogenase to pyruvate oxidase. Interestingly, a similar trend involving the induction of pyruvate oxidase activity has been experimentally observed in *L. mesenteroides* when the culture condition is changed from anaerobic to aerobic mode^49,51,53^. Taken together, these results imply that the dependence of enzyme activities corresponding to the observed metabolic shifts upon oxygen supply can be solely explained on the basis of prevailing intracellular capabilities to restore redox balances.

### MLF is unaffected by the participation of pyruvate and oxaloacetate as reaction intermediates

**Figure 5:**
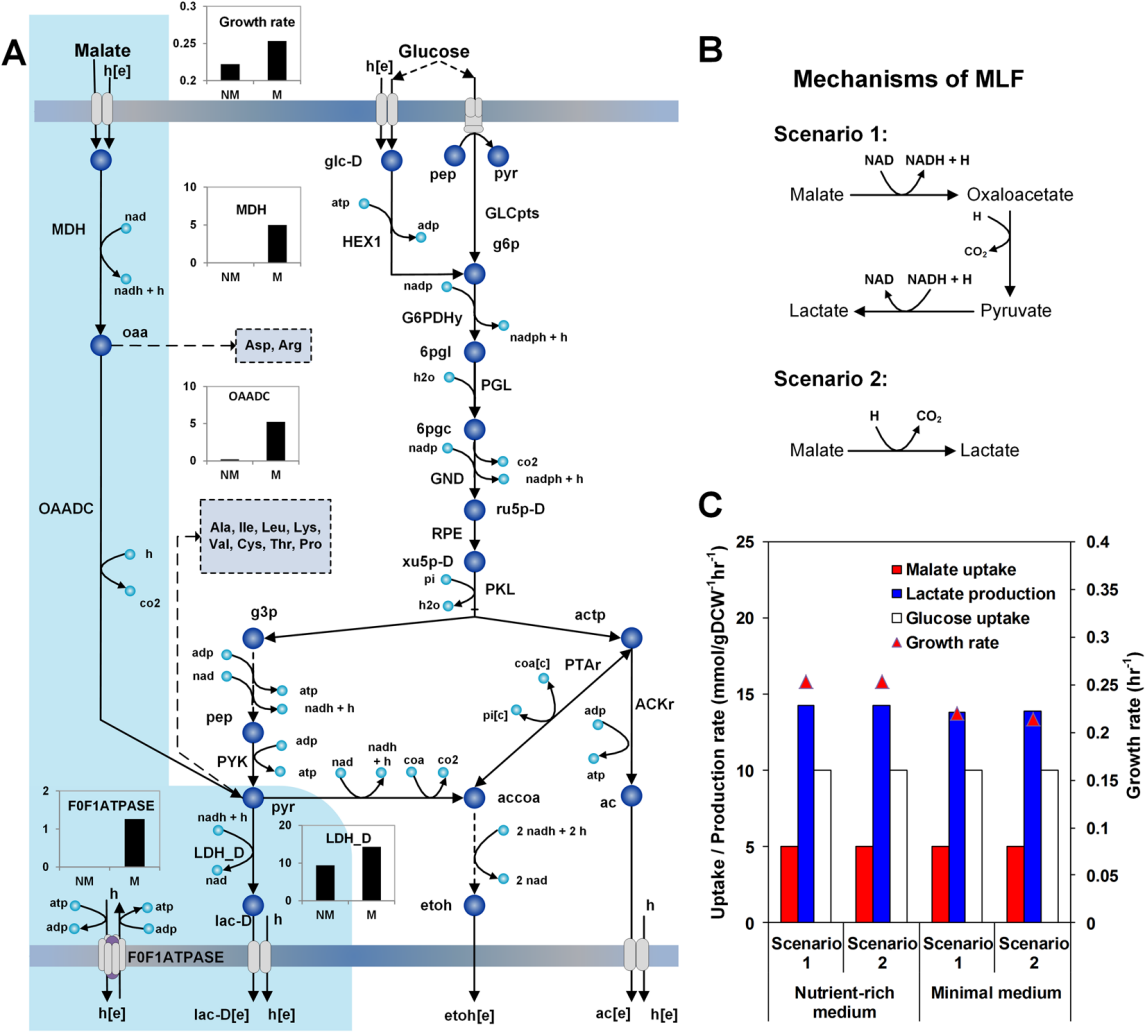
Effect of different malolactic fermentation (MLF) mechanisms on the MLF flux. (**A**) MLF and its relationship with the carbon central metabolism, amino acid biosynthesis and energy generation. Bar graphs provide the flux values (mmol gDCW^−1^ hr^−1^) of reactions part of MLF and growth rates with the supply of L-malate (M) and with no supply of L-malate (NM). (**B**) Two of the mechanisms of MLF that have been proposed by previous studies. (**C**) Effect of the differences in enzymatic routes of the two mechanisms on the malolactic flux and growth in minimal and nutrient rich media.

The low energetic yield of the PKP when compared to EMP pathway imposes severe energy limitations in *L. mesenteroides*. In this regard, MLF is an important alternate pathway used by *L. mesenteroides* for overcoming energy limitations under low-nutrient or unfavorable growth conditions (e.g., wine fermentation^13^, cucumber and cabbage fermentations^3^). During the MLF phase of wine production, malic acid is converted into lactic acid, thus reducing the excess acidity and enhancing organoleptic properties of wine. However, it is still challenging to control the rate of MLF during wine fermentation, which further prompts for better understanding of the underlying mechanism. A previous study has proposed the conversion of malate to lactate by malolactic enzyme in a single decarboxylation mediated reaction^54^. Although lactate is the only final product of the active enzyme complex, the requirement of NAD as an essential cofactor in the reaction indicates the possible participation of oxaloacetate and pyruvate as intermediates^13^. Hence, the MLF may be represented as the combination of three reactions catalyzed by malate dehydrogenase (EC 1.1.1.37), oxaloacetate decarboxylase (EC 4.1.1.3) and lactate dehydrogenase (EC 1.1.1.28) (Fig. 5A **)**. While the exact combination of MLF is still debatable^55^, it is not clear whether the involvement of the central metabolic intermediates, pyruvate and oxaloacetate, in the three-step MLF would affect the flux towards malate. Therefore, we analyzed the cellular phenotype under both scenarios, i.e., one-step and three-step MLF (Fig. 5B). Counterintuitively, both cases did not significantly affect the overall flux through the malolactic reaction(s), under both minimal and nutrient rich media (Fig. 5C). This analysis showed that instead of utilizing pyruvate and oxaloacetate for the synthesis of biomass precursors such as amino acids, *L. mesenteroides* invariably converts the entire assimilated malate to the fermentative by-product, lactate, suggesting a stronger need for regenerating redox equivalents in the organism.

Furthermore, we evaluated the role of MLF in the cell growth by characterizing metabolic flux states obtained from the model simulation^13,56^. The flux distributions determined with and without the malate supply revealed that while malate does not contribute to the anabolic carbon pool, a single proton excluded via lactate efflux transporter generates additional ATP for higher growth rate **(**Fig. 5C **)**. We observe that the ATP yield due to malate consumption increases by 0.25 moles per mole of malate, which is resulted from a reduced flux through F_0_F_1_-ATPase. This value is close to the energy gain due to malate uptake seen in *Oenococcus oeni*, 0.28 moles of ATP per mole of malate^56^, asserting MLF as one of the alternative energy conserving processes in heterofermentative LAB.

### Existence of proton symporter-dependent glucose-phosphotransferase system activity in obligate heterofermentative LAB?

**Figure 6:**
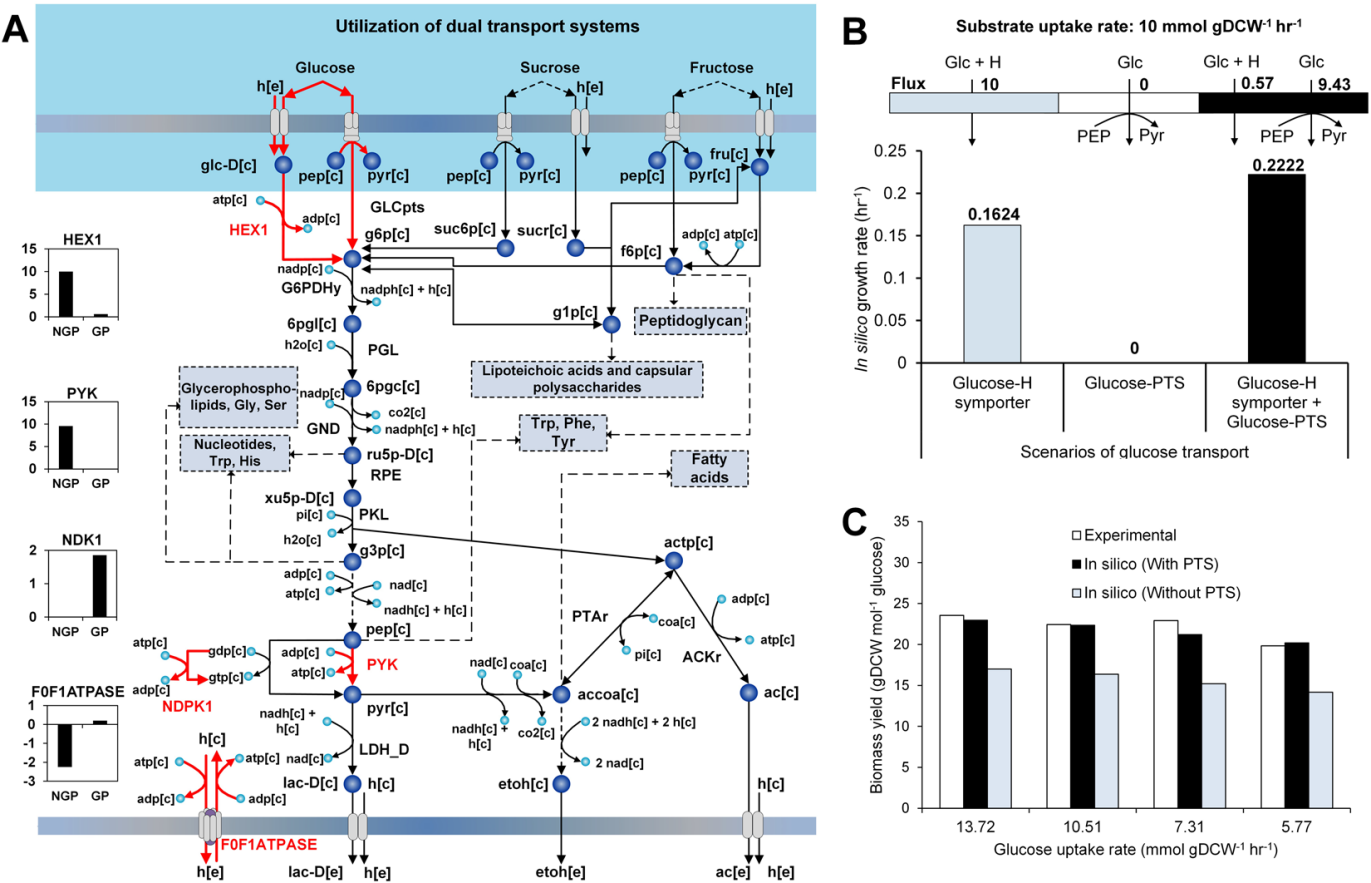
Utilization of phosphotransferase system (PTS) for glucose uptake. (**A**) Sugar PTS systems and their relationship to the carbon central metabolism and biomass precursor biosynthesis. Reactions that carry differential flux with and without the usage of PTS for glucose uptake are represented by ‘red’ lines and the corresponding fluxes are provided as bar graphs (**B**) Different scenarios of glucose transport via usage of PTS and proton-symporter, and the corresponding growth rates. (**C**) Bar graph showing comparison of experimentally obtained biomass yields from glucose with the *in silico* values simulated with and without glucose-PTS.

Energetics of sugar uptake systems play an important role in the cell growth of most bacteria as they can consume significant proportion of the total ATP generated. For example, sugar-phosphotransferase systems (PTS, Fig. 6A), compared to other active transport systems, confer additional benefit to the organism by coupling nutrient transport to substrate level phosphorylation, thereby increasing its ATP efficiency. Previous reports have claimed that *L. mesenteroides* and other heterofermentative bacteria may not utilize PTS for glucose uptake since the single molecule of phosphoenolpyruvate (PEP) produced per molecule of glucose catabolized via PKP would be used up for its own transport^15,57^. As a consequence, no PEP would be leftover for the biosynthesis of aromatic amino acids and N-acetylmuramic acid that are required for cell wall synthesis. However, the presence of putative genes encoding glucose-PTS components in *L. mesenteroides* (LEUM_0507, LEUM_0508 and LEUM_0901) as annotated in TransportDB database^35^ allowed us to analyze the possible cellular phenotype of *L. mesenteroides* growing on glucose with and without a glucose-PTS transporter. Accordingly, we tested three possible scenarios of glucose transport as illustrated in Fig. 6B. The first scenario involves a simple glucose-proton symporter, a relatively less energy efficient transport in bacteria that use glucose-PTS transporter. The second one exclusively uses glucose-PTS transporter, which turns out to be infeasible because of the reason described earlier. The last scenario utilizes both transporters in combination. Noticeably, maximum growth occurs when a small proportion of glucose is consumed via glucose-proton symporter towards PEP synthesis, while the rest is transported via PTS for net ATP production (Fig. 6B).

Model simulations showed that the usage of glucose-PTS results in significantly increased biomass yield from glucose i.e., the ratio of growth rate to glucose uptake rate **(**Fig. 6C **)**. Therefore, we compared the experimentally determined biomass yields for *L. mesenteroides* anaerobically grown on glucose as sole carbon source^48^ with the simulated yields for the two scenarios, with and without glucose-PTS usage. Figure 6C clearly shows that the experimentally observed biomass yields for both cases are consistent with the scenario involving glucose-PTS. In conjunction to this, a report showing glucose-PTS activity in a different obligate heterofermenting LAB, *O. oeni* ^58^, very much imply that all obligate heterofermentative LAB are capable of utilizing PTS for transporting glucose.

### Integrated transcriptomic analysis reveals regulatory bias towards fluxes correlated to mannitol production

Sucrose is known to support higher growth rate in *L. mesenteroides* compared to the monosaccharides, glucose and fructose^49^ although the molecular basis for this observed difference is still unclear. Therefore, we initially performed constraint-based flux analysis to evaluate effects of two carbon sources (sucrose and glucose) on metabolic utilization. As expected, the resultant flux distributions did not reveal any significant differences apart from the initial carbon assimilatory pathways, which motivated us to explore metabolic regulation by profiling the gene expression of *L. mesenteroides* grown in sucrose or glucose as sole carbon source (see Methods). Subsequently, we carried out metabolic subsystem-wise enrichment analysis using *i*LME620. Interestingly, the presence of sucrose in the culture medium induced the upregulation of genes encoding sucrose phosphotransferase system (91-fold) and sucrose-6-phosphate hydrolase (72-fold). However, no such induction of genes involved in the substrate assimilation was found for the glucose uptake. In addition to the sugar uptake, several metabolic pathways related to biomass precursor production, including fatty acid biosynthesis, biotin biosynthesis, undecaprenyl diphosphate biosynthesis, and the overall uptake transporters were upregulated (Table 1). Moreover, the energetically costlier purine biosynthesis was selectively down-regulated upon sucrose supply, while the pyrimidine biosynthesis was simultaneously up-regulated, however, the reasons for such differential expression remains unknown. Although the upregulation of biomass precursor biosynthetic pathways observed from the enrichment analysis clearly suggest that growth on sucrose predisposes *L. mesenteroides* for higher biomass production capabilities, the nature of driving force(s) behind such capabilities remain elusive.

**Table 1:** Metabolic subsystems part of *i*LME620 differentially expressed among glucose and sucrose uptake conditions.

Subsystem	Effect in sucrose with glucose as reference	P-value differential expression (Wilcoxon signed-rank test)
Biotin metabolism	Upregulated	0.0151
Fatty acid biosynthesis	Upregulated	0.00000195
Pyrimidine metabolism	Upregulated	0.0234
Starch and sucrose metabolism	Upregulated	0.0400
Undecaprenyl diphosphate biosynthesis	Upregulated	0.0024
Transport reactions	Upregulated	0.0401
Lysine biosynthesis	Downregulated	0.0029
Purine metabolism	Downregulated	0.0039

P-values (<0.05) were obtained using Wilcoxon signed rank test.

**Figure 7:**
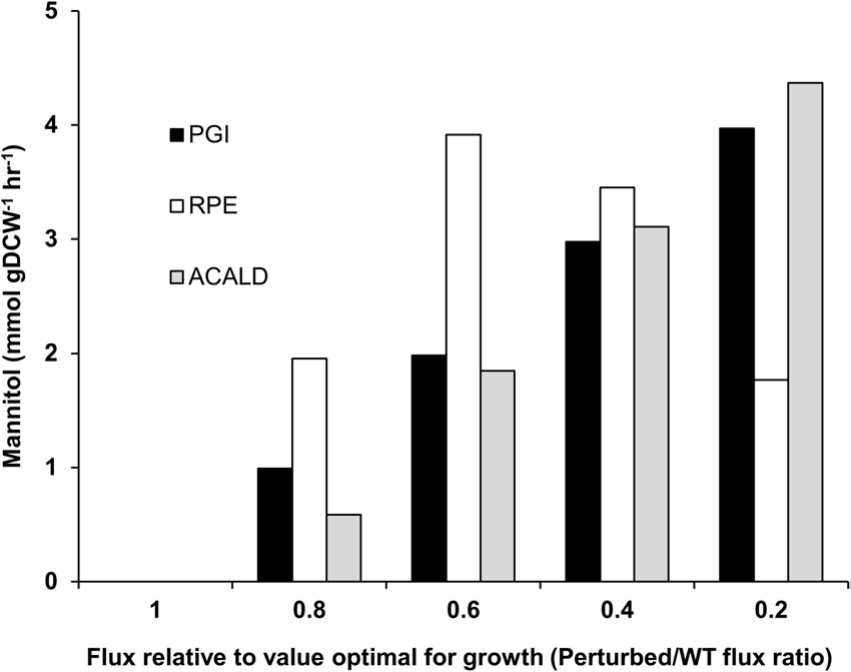
Control of mannitol production by transcriptionally regulated enzymes. Effect of flux through PGI (glucose-6-phosphate isomerase), RPE (ribulose 5-phosphate 3-epimerase) and ACALD (acetaldehyde dehydrogenase) on mannitol production. The flux through each of these reactions was varied from a value optimal for growth to a minimum feasible value determined by flux variability analysis and the corresponding mannitol production fluxes were recorded. WT flux: Wild type flux or flux optimal for growth.

We then examined the correlation between gene expression and metabolic fluxes, and identified the transcriptionally, post-transcriptionally, or metabolically regulated reactions under sucrose to glucose growth transition by resorting to a method that takes into account the direction of change of the *in silico* fluxes derived from a constraint-based sampling procedure and the corresponding changes to gene expression levels (see Methods). Table 2 summarizes the top-scoring (probability > 0.9) reactions in each category. We could not derive any meaningful inferences from the post-transcriptionally and metabolically regulated enzymes. However, through additional simulations, we observe that fluxes through reactions catalysed by all the three enzymes identified as transcriptionally regulated, including glucose-6-phosphate isomerase (PGI), acetaldehyde dehydrogenase (ACALD) and ribulose 5-phosphate 3-epimerase (RPE), were negatively correlated to mannitol production **(**Fig. 7 **)**. This result is interesting as mannitol is the preferred by-product of *L. mesenteroides* to relieve redox imbalances during fermentative growth conditions, such as the early stages of sauerkraut production^3^.

**Table 2:** List of transcriptionally, post-transcriptionally and metabolically regulated enzymes during glucose-sucrose growth transition.

Regulatory level	Enzymes	Probability scores
Transcriptionally regulated enzymes	Aldehyde dehydrogenase	0.98
	Glucose-6-phosphate isomerase	0.99
	Ribulose 5-phosphate 3-epimerase	0.90
Post-transcriptionally regulated enzymes	Acylphosphatase	0.98
	D-alanine-D-alanine ligase	0.92
	Argininosuccinate lyase	0.97
	ABC transporter of L-proline	0.99
	GTP phosphohydrolase	0.99
	Xanthine phosphoribosyltransferase	0.96
	Xanthosine ribohydrolase	0.98
Metabolically regulated enzymes	Water channel	0.99
	Phosphoglycerate kinase	0.95

Probability scores (>0.9) for each regulatory category were calculated using the method developed by Bordel *et al*.^74^.

## Discussion

In this study, we presented the first genome-scale metabolic model of obligate heterofermentative LAB, *L. mesenteroides* ssp. *mesenteroides*, for better understanding of crucial metabolic modules potentially influencing its industrial and probiotic applications. The *in silico* growth simulations performed using the model revealed negligible capability of *L. mesenteroides* to utilize amino acids as energy source unlike other LAB groups^59^. Such observation is presumably due to the absence of two important processes, the ATP-forming deiminations and proton-motive force generating amino acid decarboxylations, which contribute to energy generation using amino acids in other LAB. They are also known to be involved in intracellular pH buffering^59^. The absence of such enzymes in *L. mesenteroides* may therefore explain the relatively low tolerance of the organism to acidic condition. In addition, the negligible amino acid utilization for energy generation explains their existence in many natural ecological niches such as green vegetables or silage^43^. Besides, it is suggested that, in an association with the gut microbiota, protein-rich diets may lead to the dominance of other LAB over *L. mesenteroides*.

The knowledge derived from model reconstruction and curation processes enabled us to speculate some unknown metabolic functionalities in *L. mesenteroides*. For example, with the exceptions of glutamine and valine, the amino acid auxotrophy predictions were consistent with single-amino acid omission experiments^40^. The glutamine discrepancy could be attributed to the presence of glutamine synthetase (GLNS) (gene locus id: LEUM_0717) in the model, which can synthesize glutamine using glutamate. However, literature evidences suggest possible *in vivo* repression of GLNS by GlnR, a transcriptional regulator of nitrogen metabolism whose repressor activity has been observed in low-GC containing Firmicutes, including *Lactococcus lactis* ^60^ and *Streptococcus pneumonia* ^61^ under nitrogen excess condition. Hence, with a refined constraint on GLNS repression, we could replicate the glutamine auxotrophy successfully. The *in silico* predictions identified valine as non-essential in contrast to the single-amino acid experiments which showed it to be essential. Such discrepancy could possibly arise due to the limitations of constraint-based modeling approach which does not capture the transcriptional and translational regulation; although *L. mesenteroides* has ORFs encoding for all the genes in valine biosynthetic pathway as accounted in *i*LME620, some of them could be impaired at translational or transcriptional levels, and thus rendering the pathway inactive.

The obligate heterofermentative LAB possess distinct metabolic pathways to generate/conserve energy. Here, we elucidated two relevant metabolic behaviors to alleviate energy limitations by proposing new hypotheses. Firstly, we suggest that the fate of L-malate consumed via the MLF is independent of the intermediate steps **(**Fig. 5A **)**. We ascribe this observation to a substrate channeling-like effect induced by the cellular redox environment, wherein surprisingly the two central metabolic intermediates, i.e. oxaloacetate and pyruvate, form lactate rather than being diverted towards the synthesis of biomass precursors. Existence of such channeling mechanism is further supported by the thermodynamic analysis of MLF: the first step converting malate to oxaloacetate has a large positive Δ_r_ *G*′° (>30 kJ/mol at pH ≤ 7)^62^, indicating the channelling of malate towards lactate formation as the best possible scenario to overcome such thermodynamic limitation. Overall, this exemplifies the dominance of redox state as a factor controlling cellular processes in *L. mesenteroides*. Secondly, we highlighted the possibility of PTS-based glucose uptake in *L. mesenteroides*. Figure 6B clearly shows that most of the glucose is metabolized through PTS to enhance the overall ATP production while only a minor portion of flux through the glucose-proton symporter is used to satisfy the biosynthetic demands of certain biomass precursors such as PEP. Hence, the utilization of PTS for glucose uptake and subsequent dissimilation of carbon flux via PKP should strictly involve its concurrent operation with alternate transporter systems, such as the glucose-proton symporter. Apart from the experimental evidences supporting this hypothesis **(**Fig. 6C **)**, discussed earlier, we also observe significant expression of the genes encoding components of glucose-PTS along with the putative glucose-permease, *glcU* in cells grown on glucose (top 10% of the highly expressed genes; see Supplemental data 1) indicating plausible glucose-PTS and glucose permease activities in *L. mesenteroides*. Additionally, we also find that the usage of PTS for glucose transport has significant effect on some of the central carbon metabolic fluxes, including those of hexokinase (HEX1), pyruvate kinase (PYK), nucleoside-diphosphate kinase (NDK) and F_0_F_1_-ATPase **(**Fig. 6A **)**. Interestingly, PYK and NDK fall under the CcpA regulon for *L. mesenteroides* and other Firmicutes^63^. Note that the transcriptional regulator, CcpA, has been known to repress glucose-PTS and NDK while activating PYK^64,65^. The flux changes of NDK and PYK during the cell growth with and without PTS usage, as depicted in Fig. 6A, indeed follow a trend that is consistent with the regulation detailed above, providing additional clues about the predisposal of the metabolic and regulatory structure in this LAB to the usage of glucose-PTS. Overall, these results suggest that although PEP availability could be a limiting factor for the *in vivo* glucose-PTS activity, *L. mesenteroides* could theoretically operate this transporter in a coordinated manner to achieve higher net ATP and growth yield.

The integrative analysis of transcriptome data allowed us to delineate the metabolic behaviour and regulation relevant to the higher growth rate observed in sucrose as compared to glucose: (i) upregulation of the genes in sucrose assimilation, (ii) net upregulation of genes for biomass precursor biosynthesis, and (iii) downregulation of PGI, ACALD and RPE which are negative flux control points of mannitol production (Fig. 7), thereby favouring mannitol production. Although the fructose component of sucrose could be used for the production of energy and biomass precursors, it can be partly diverted towards mannitol biosynthesis. These evidences thus suggest the existence of redox imbalances and a transcriptional regulatory bias towards metabolic pathways relieving these imbalances in *L. mesenteroides*.

In conclusion, our work explained several features unique to *L. mesenteroides*, and provided a reliable *in silico* chassis that comprehensively represents obligate heterofermentative metabolism. Discovery of less studied regulatory mechanisms such as the substrate channeling during MLF described here could aid in robust and efficient design of heterologous pathways^66^. Evidently, the intracellular redox state has been observed as the major phenotype governing factor in *L. mesenteroides*. Therefore, the future endeavours to evaluate metabolic capabilities of this LAB should take into account the importance of achieving redox state desirable for specific industrial and therapeutic applications.

## Methods

### *L. mesenteroides* culture

Chemostat cultivations of *L. mesenteroides* ssp. mesenteroides ATCC 8923 were performed in an anaerobic culture system. Briefly, cells were grown using a chemically defined medium (CDM) developed earlier^40^, under carbon limited conditions at a dilution rate (D) of 0.1 h^−1^, with no oxygen in the bioreactor inlet air. The total inlet gas (N_2_) flow was controlled by mass flow meters (Bronkhorst High-Tech, Netherlands) at 1.5 vvm. The pH, stirring speed and temperature were maintained at 6.8 (using 0.5 N NaOH), 700 rpm and 30 °C, respectively. Biomass harvested from the chemostat culture was subsequently used to analyze the macromolecular composition (see next section).

### Biomass composition analysis

Drafting a concise biomass assembly equation representing the overall cellular composition of *L. mesenteroides* is an important prerequisite for analysing the intracellular metabolism at exponential phase of batch culture or at steady state in chemostat, where the primary aim of cell is to grow. Therefore, in order to formulate such biomass equation, we experimentally measured the total protein, carbohydrates, RNA and DNA content, and the detailed amino acid composition of *L. mesenteroides* from lyophilized cells harvested from steady state chemostat culture. In each case, at least three independent samples, from two independent cultivations, were analyzed.

Elemental analysis was performed using 1 mg of a lyophilized biomass. After adding 1 mg of V2O5, the samples were subjected to 1000 °C in an oven. The volatile compounds thus released from the sample were measured using an Elemental Analyzer (NA2000 ThermoFisher, USA).

Total carbohydrates were determined by the phenol method as described earlier^67^. Total protein content was determined by means of the BCA method^68^. Protein concentration was calculated using bovine serum albumin (BSA) as standard. The total lipid content was determined after methanol–chloroform extraction as described elsewhere^69^. Genomic DNA (gDNA) and total RNA was extracted from *L. mesenteroides* grown in the CDM at 30 °C under anaerobic conditions until the cell number reached 10^8^–10^9^/ml. The chromosomal DNA was extracted from the cells using a gDNA prep kit (SolGent, Korea) and RNA extracted using a RNA prep kit (MACHEREY, Germany). Quantification and purity were measured by nanodrop (Epoch, BioTek Laboratory, Shoreline, WA, USA).

Amino acid composition of the proteins extracted from *L. mesenteroides* cells was determined using the amino acid analyzer (Biochrom 30; using the Biochrom EZ Chrom software, for quantification) using a cation exchange chromatography column and post-derivatization with ninhydrin as described previously^70^. Nucleotide composition of DNA was calculated using the GC content of *L. mesenteroides* genome (37.8%)^32^. Total glycerolipid, phospholipid and peptidoglycan content, and fatty acid composition were accounted based on bibliographic information (Supplemental data 1). The stoichiometric coefficients calculated for biomass precursors are represented in terms of mmol gDCW^-1^ (milli moles per gram of dry cell weight) in the equation. Dry cell weight was obtained by weighing the lyophilized cells. Additional details regarding the formulation of biomass equation are provided in the Supplemental data 1.

### Metabolic network reconstruction

The genome scale metabolic model of *L. mesenteroides* was reconstructed based on the genome annotation^32^ and information compiled from various databases and literature, following the well-established protocol^31^. Initially, a draft metabolic network was reconstructed by reconciling the *L. mesenteroides* specific metabolic and transport reactions collected from KEGG^34^, MetaCyc^33^ and TransportDB^35^ databases. Duplicate and generic reactions were subsequently eliminated from the draft reconstruction using EC numbers and NCBI locus tags as common identifiers. The reconstruction content was then manually curated for reaction directionality, elemental and charge balancing of individual reactions. We employed the thermodynamic data generated by earlier studies^37–39^ to assign reaction directionality in the current model using the approach proposed earlier^38^. Note that those reactions with conflicting information on directionality were assigned to be reversible. The directionality of the reactions, for which no information was available, were determined based on heuristic information available in published literature and/or from biochemical databases such as Brenda^71^ and MetaCyc^33^. Metabolic dead-ends were then identified using the GapFind algorithm^36^ and were subsequently filled in by the addition of new reactions from other organisms based on literature evidences. Here, in order to substantiate the existence of such reactions in *L. menesteroides*, protein sequence homology of the corresponding enzymes were performed prior for their inclusion. Additionally, certain non-gene associated reactions were included in the reconstruction based on either direct or indirect biochemical evidences from the literature. Finally, the gene-protein-reaction (GPR) associations were manually added to each reaction based on the enzyme subunit and isoenzyme information obtained from the biochemical databases.

### Constraint-based flux analysis

Constraint-based flux analysis was employed to analyze the metabolic phenotype of *L. mesenteroides* while growing on a nutrient rich medium. The biomass equation was maximized while simultaneously constraining the uptake rates of various amino acids and vitamins, and secretion rates of fermentation by-products based on a previously published study^40^. The optimization problem can be mathematically represented as follows:

1 $${\rm{\max }}\,Z=\sum _{j}{c}_{j}{v}_{j}$$

2 $${\rm{s}}{\rm{.t}}.\sum _{j}{S}_{ij}{v}_{j}=0\forall \,{\rm{metabolite}}\,{i}$$

3 $${v}_{j}^{\min }\le {v}_{j}\le {v}_{j}^{\max }\,\forall \,{\rm{reaction}}\,j$$

where *S*_*ij*_ is the stoichiometric coefficient of metabolite *i* participating in the reaction *j*; *v*_*j*_ represents flux through the reaction *j*; *v*_*j*_^*min*^ and *v*_*j*_^*max*^ respectively represent the lower and upper bounds on the flux through the reaction *j*; and *Z* is the biomass objective, where *c*_*j*_ denotes the relative contribution of each metabolic reaction to the biomass formation. Flux variability analysis and flux-sum analysis were performed extending the formulation shown above as described earlier^52,72^. Briefly, the turnover rate of metabolite *i* (*ϕ*_*i*_) obtained using flux-sum analysis is given by

4 $${\varphi }_{i}=\frac{1}{2}\sum _{j}|{S}_{ij}\cdot {v}_{j}|$$

Amino acid auxotrophic simulations were performed by setting each amino acid uptake rate to zero, individually, while maximizing the growth cellular objective. In this study, amino acid auxotrophy is defined by the condition of > 90% growth reduction due to exclusion of corresponding amino acid from the media. All constraint-based flux analysis simulations including gene-essentiality analysis were performed using COBRA toolbox^72^ with Gurobi5 (http://www.gurobi.com) as optimization solver.

### Thermodynamic analysis of metabolic pathways

In order to perform pathway-wise thermodynamic analysis and to calculate the transformed Gibbs free energy changes of the corresponding metabolic reactions under physiologically relevant pH (7.2), ionic strength (0.1 M) and intracellular metabolite concentrations (1μM-10mM), we used the web-based tool, eQuilibrator (http://equilibrator.weizmann.ac.il). This tool employs a database containing thermodynamic information along with an extended version of the group contribution method to perform the Gibbs free energy calculations^73^.

### Sensitivity analysis

The sensitivity of cell growth to variation in maintenance energy costs (GAM and NGAM) and the macromolecular composition was evaluated using two different measures. Firstly, each parameter was individually adjusted within a ± 50% error range, and the corresponding effect on the growth objective was determined. Secondly, sensitivity coefficients were calculated as the ratio of change in the growth objective to a unit change in the stoichiometric coefficient of each macromolecular component in the biomass equation.

### Microarray hybridization and expression data analysis

The gene expression of *L. mesenteroides* was profiled while growing on sucrose (S) and glucose (G) as sole carbon source in MRS broth. cDNA samples were prepared from the total RNA extracted using a RNA prep kit (MACHEREY, Germany) from exponentially grown cells of S and G groups in two biological replicates. The oligonucleotide probes (35–40 mers, 2033 probes) for 1871 genes, designed based on the genome sequence of *L. mesenteroides* (Genbank accession no. CP000414), were synthesized and printed onto the CombiMatrix 4 × 2 K custom array platform (Macrogen Inc., Korea). The probe design, spotting and analysis were conducted by Macrogen. Local-pooled-error (LPE) test and fold change were applied using R 2.4.1 software to determine and evaluate the significance of each gene’s differential expression in S and G groups.

### Analysis of transcriptomic data for differentially expressed pathways using *i*LME620

The gene-expression data with coefficient of variance (CV) < 0.3 (less than 30% variance between replicate data points) from the quantile normalized microarray datasets of the S and G groups were employed to identify the differentially expressed pathways between sucrose and glucose uptake conditions. To do so, the gene expression values were mapped to the corresponding locus tags in *i*LME620 using the gene-protein-reaction associations (GPRs). Gene-expression fold-change values were converted into reaction fold-change. Lowest fold change value between two or more genes with “AND” relationship was considered to account for the proportional fold-changes between the subunits of an enzyme complex required to effectuate the overall reaction fold-change. For reactions associated with genes having “OR” relationship, the fold-change values were added to account for the additive nature of intracellular enzyme concentrations. Significance of differential expression of various subsystems in S and G uptake conditions was determined using two-sided Wilcoxon signed rank test.

### Identification of transcriptionally, post-transcriptionally and metabolically regulated reactions

A Z-score-based approach was used to identify the transcriptionally, post-transcriptionally and metabolically regulated reactions^74^. First, the flux solution space of *L. mesenteroides* growing on S or G was sampled using the OptGpSampler^75^ implemented in COBRA toolbox. The significance of change between the expression and flux of each reaction was then Z-scored using the formula below:

5 $${Z}_{i}^{E}=\frac{mean(E{2}_{i})-mean(E{1}_{i})}{\sqrt{Variance(E{2}_{i})+Variance(E{1}_{i})}}$$

6 $${Z}_{i}^{F}=\frac{mean(F{2}_{i})-mean(F{1}_{i})}{\sqrt{Variance(F{2}_{i})+Variance(F{1}_{i})}}$$

where *Z*_*i*_^*E*^ and *Z*_*i*_^*F*^ are the Z-scores for expression and flux of reaction *i*, respectively. *E1*_*i*_ and *E2*_*i*_ are the expression intensity values and *F1*_*i*_ and *F2*_*i*_ are the flux values of reaction *i* in condition 1 (S) and condition 2 (G), respectively. Note that 5000 sample points using 500 steps between each point was required for good convergence, as suggested earlier^74^. OptGpSampler^75^ implemented within COBRA toolbox was used to obtain sample points for both S and G conditions. Subsequently, the Z-scores were used to calculate the probabilities of correlation between the flux and gene expression of reaction. Briefly, a reaction is said to be transcriptionally regulated if the product of the probability of expression change of a reaction and the probability of the flux of the reaction changing in the same direction is significant. A reaction is considered metabolically regulated if the probability of no change in its expression level but change in flux is significant. And, the reaction is post-transcriptionally regulated if the probability of change in its expression level not leading to a change in its flux is significant.

## Electronic supplementary material

Supplemental Figures

Dataset 1

Dataset 2
