# Supplementary material for: Genome-scale modeling and transcriptome analysis of Leuconostoc mesenteroides unravel the redox governed metabolic states in obligate heterofermentative lactic acid bacteria
Source: Sci Rep. 2017 Nov 16;7:15721. doi: 10.1038/s41598-017-16026-9 (PMC5691038; doi:10.1038/s41598-017-16026-9)
Supplement: Supplementary file 1 — Supplemental Figures [file 41598_2017_16026_MOESM1_ESM.doc]

# Supplementary Information

**Genome-scale modeling and transcriptome analysis of *Leuconostoc mesenteroides* unravel the redox governed metabolic states in obligate heterofermentative lactic acid bacteria**

Lokanand Koduru, Yujin Kim, Jeongsu Bang, Meiyappan Lakshmanan, Nam Soo Han and Dong-Yup Lee


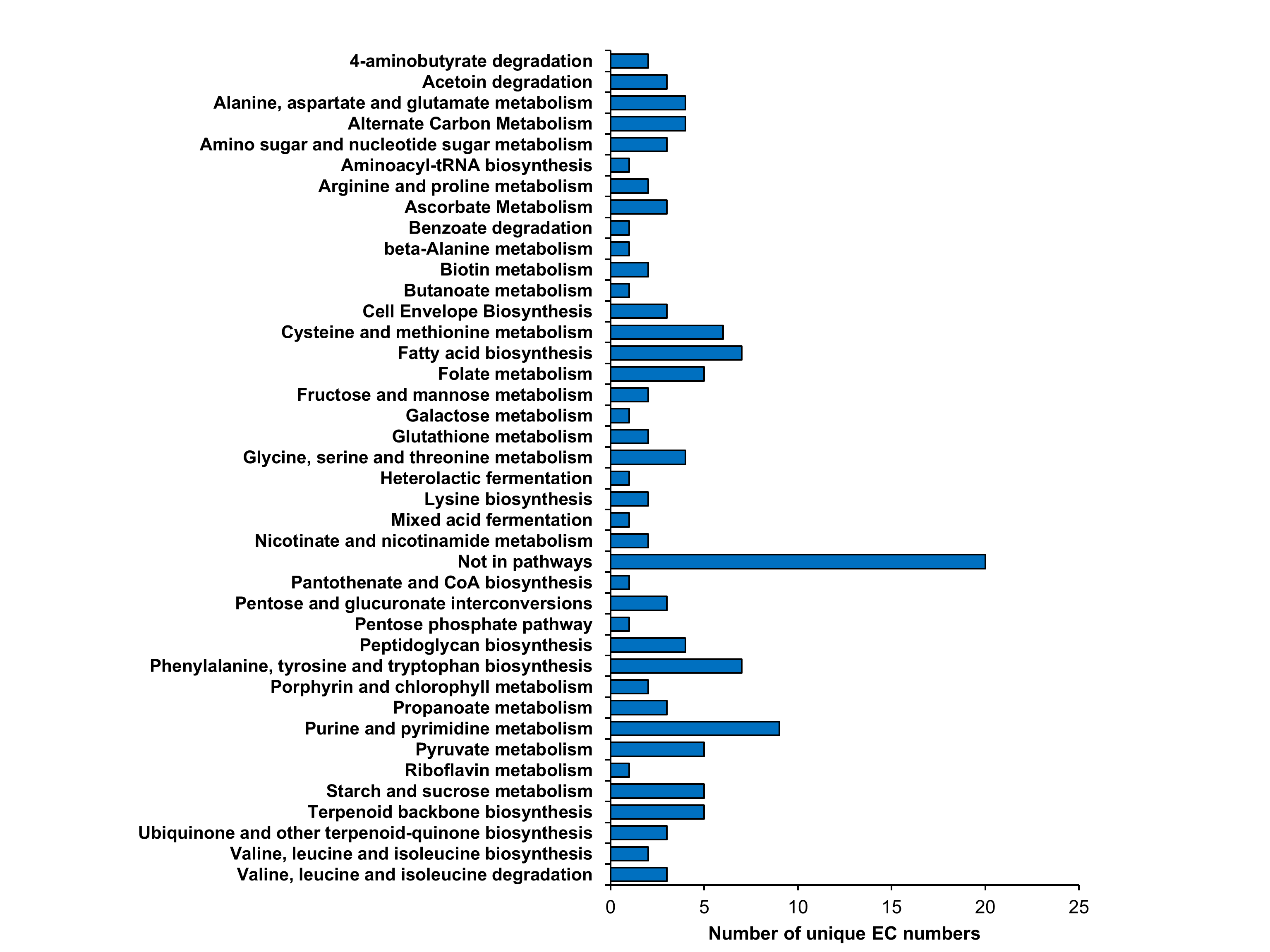


**Supplemental Figure 1:** Distribution of unique EC numbers across various subsystems in *L. mesenteroides*


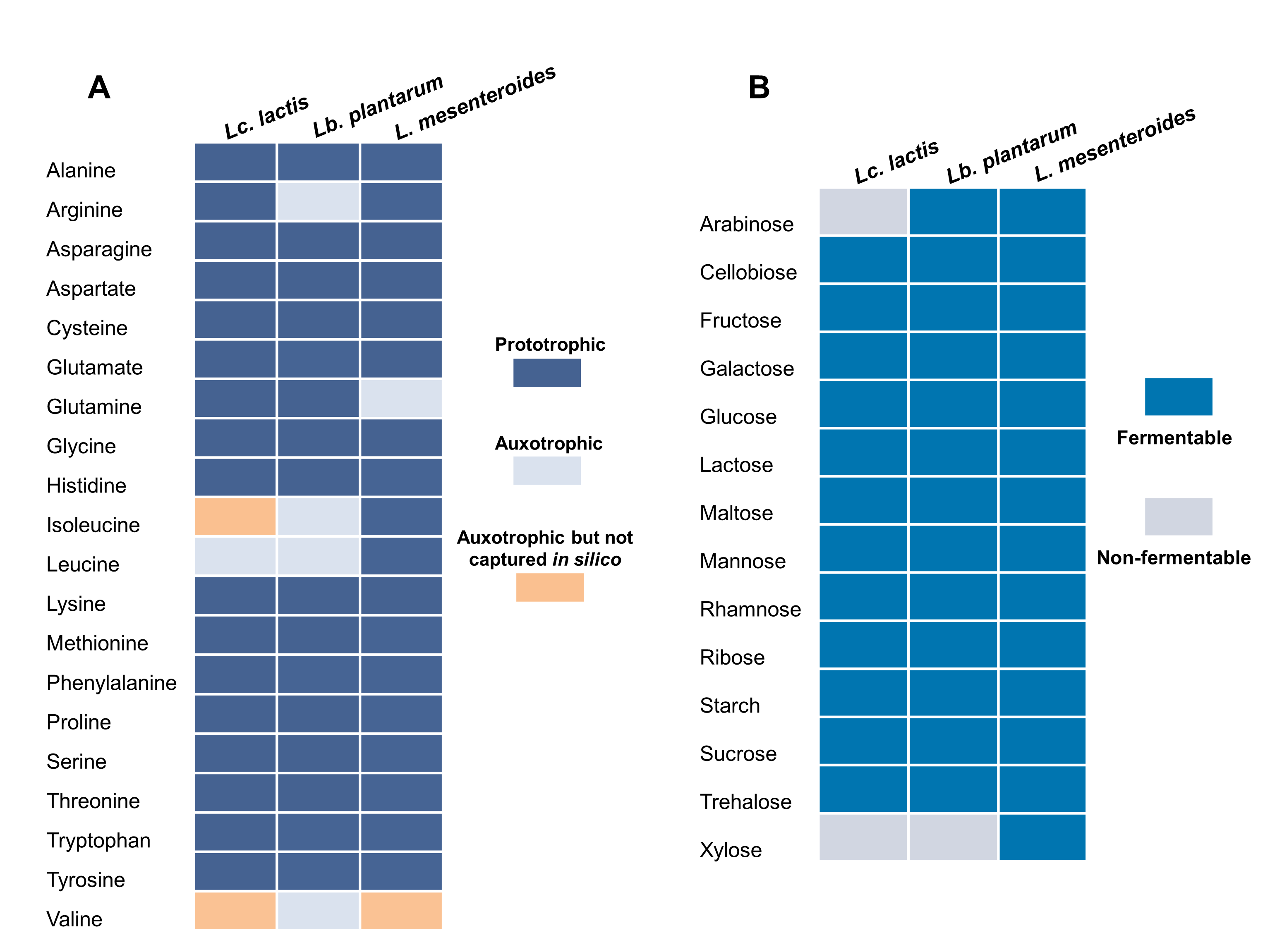


**Supplemental Figure 2:** Comparison of amino acid auxotrophy and fermentable sugar phenotyping among different LAB


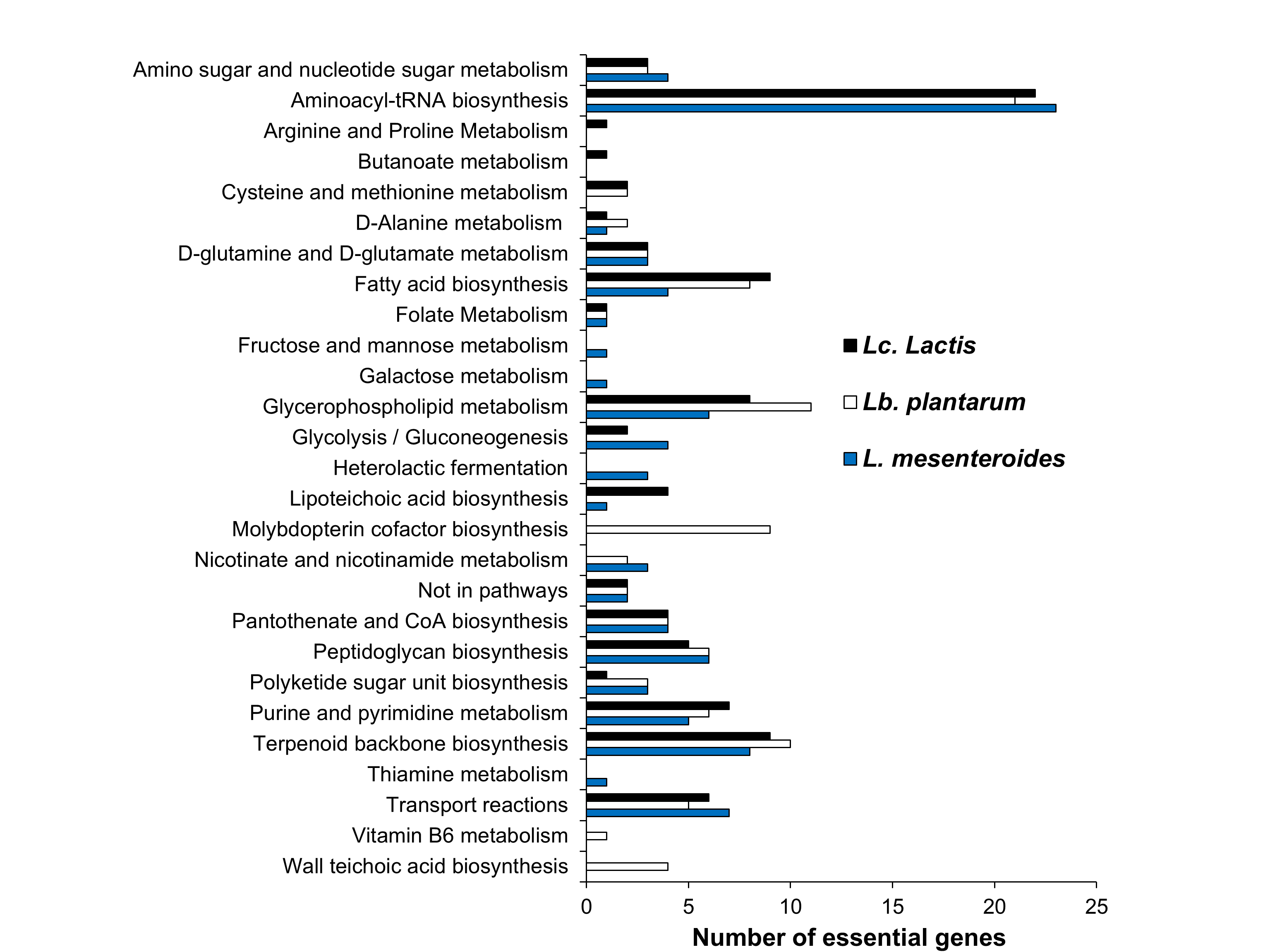


**Supplemental Figure 3:** Comparison of essential gene distribution among different LAB
